# Comment on the article: “Microvascular effects of intravenous esmolol in patients with normal cardiac function undergoing postoperative atrial fibrillation: a prospective pilot study in cardiothoracic surgery”

**DOI:** 10.1186/s13054-019-2412-y

**Published:** 2019-04-16

**Authors:** Ye Ying Zheng, Fei Guo, Bin Lu, Qiang Li

**Affiliations:** Department of Anesthesiology, Zigong Fourth People’s Hospital, Zigong, Sichuan Province China

Dear Editor,

We read with great interest the study by Fornier and colleagues [1] in which they demonstrated that intravenous application of esmolol reversed microvascular dysfunction, but not hemodynamic instability, in patients with postoperative atrial fibrillation. We appreciate the contribution that this prospective pilot study makes to the field. At the same time, we caution that the results should be interpreted with care in light of some methodological issues.

The authors stated: “A rapid arterial occlusion of the upper limb was provoked by inflation of the pneumatic cuff at 50 mmHg above the systolic arterial pressure, until either the tissue oxygen saturation (StO_2_) value decreased to 40% or for a maximal period of 10 min”. It was therefore possible that the minimum StO_2_ value in some patients was higher than 40% after 10 min in the vascular occlusion test, which may have led to faster tissue oxygen re-saturation speed [2]. Such differences in re-saturation speed were not controlled for in the study and so may have influenced the results.

The authors further stated that “all patients were monitored with a five-lead electrocardiogram with computerized analysis of repolarization and invasive or non-invasive arterial blood pressure”. This means that arterial blood pressure was determined using different methods among the patients, and this variation of arterial blood pressure determination may have contributed to the results. Compounding this method-related variability is the fact that atrial fibrillation patients show relatively large short-term blood pressure fluctuations because of variability in left ventricular stroke volume.

Four patients in the study were treated with norepinephrine, yet the dose was not reported. Norepinephrine affects StO_2_ [3], so dose information is needed in order to assess the risk that this treatment may have affected the results. Similarly, the study does not report whether patients received interventions against causes of elevated heart rate, such as infection, endocrine imbalance, anemia, or pulmonary embolism [4]. These interventions can interfere with the effects of esmolol, so information on their use should be included.

Studies like those of Fornier and colleagues [1] should attend to these points in order to provide the most reliable insights into improving care for patients experiencing postoperative atrial fibrillation.

## Abbreviation

StO_2_: Tissue oxygen saturation
